# Severity and factors of menopausal symptoms in middle-aged women in Gansu Province of China: a cross-sectional study

**DOI:** 10.1186/s12905-021-01531-x

**Published:** 2021-12-08

**Authors:** LiRong Wang, Rui Zhang, Ye Yang, XiaoYan Sun, BaoLi Zhang, HaiYing Zhu, XiaoRong Luo, XiaoLing Ma, XueHong Zhang

**Affiliations:** 1 The Reproductive Medicine Special Hospital of the First Hospital of Lanzhou University, Key Laboratory for Reproductive Medicine and Embryo, Lanzhou, Gansu China; 2 The First School of Clinical Medicine of Lanzhou University, Lanzhou, Gansu China; 3 The First Hospital of Lanzhou University, Key Laboratory for Reproductive Medicine and Embryo of Gansu Province, No. 1, Donggangxi Rd, Chengguan District, Lanzhou City, 730000 Gansu Province China

**Keywords:** Menopausal symptoms, Cross-sectional study, Middle-aged, China

## Abstract

**Objective:**

To investigate the severity and risk factors of menopausal symptoms in the middle-aged women in Gansu Province of China.

**Methods:**

In this cross-sectional study, a total of 7319 women (aged 40–55 years) attended the health survey in Gansu Province in China were enrolled. Information on demographics, menopausal status, reproductive history, and history of chronic diseases was collected via a structured questionnaire. Severity of menopausal symptom was evaluated by the Modified Kupperman Menopausal Index. Ordinal logistic regression model was applied to explore its risk factors.

**Results:**

A total of 7319 participants were included in present study, among them, 3606 (49.27%) had moderate or severe menopausal symptom. Compared with premenopausal women, perimenopausal and postmenopausal women have a higher mKMI score. We observed that older age, higher BMI, non-married status, longer duration of menstruation (≥ 7 days), number of pregnancy (> 3 times), longer duration of breastfeeding (> 12 months), peri- or post-menopausal status, and menopause hormone therapy was positively associated with menopausal symptom score, while higher level of family income, educational and physical activity, and history of gynecological, breast or chronic disease were negatively associated with the score.

**Conclusions:**

Numerous factors were associated with the severity of menopausal symptom among the Chinese women. Because it was gradually increased with aging, more attention is warranted to manage the menopausal symptom.

**Supplementary Information:**

The online version contains supplementary material available at 10.1186/s12905-021-01531-x.

## Introduction

Menopause is defined by the World Health Organization (WHO) as the permanent cessation of menstruation and a decrease in the levels of ovarian steroid hormones (estrogen and progesterone) [1], which is a critical period that marks the end of reproductive ability [2, 3]. More than 90% [4] women could have serious short-term and long-term menopausal syndromes, including physical such as vasomotor (hot flashes, sweating), psychological (tension, anxiety, depression), and urogenital (vaginal dryness, sexual dysfunction) symptoms. The presence of menopausal symptoms could lead to low quality of life and even cause severe mental illness [5, 6].

Increased negative psychological symptoms (anxiety and depression [7]) and decreased cognitive performance during menopausal transition could last up to the age of 60 and then get worse during the following years [8]. The risk of osteoporosis [9], hypertension [10], cardiovascular diseases [11] and metabolic disorders [12] would increase as well. The occurrence and risk factors of menopausal symptoms can vary in women with different genetic, cultural, and regional backgrounds [13]. According to a report, the number of women over 50 years in China will arise to more than 280 million and the global will grow to 1.2 billion by 2030 [14]. For women spend a third of their lives in perimenopausal and postmenopausal period [15], investigating the predictive or protective factors of the severity of menopausal symptoms may help to relieve the symptoms.

Previous studies on menopause-related health problems have been mainly conducted in Western countries [16, 17], while few studies was in China [18], especially in the northwest area where the present study was conducted. The northwest region differs in its socioeconomic and cultural characteristics to the rest of the China, which may have variations in prevalence and intensity, as well as in the determinants of menopausal symptoms.

In this cross-sectional study, we aimed to investigate the severity and risk factors of menopausal symptoms measured by mKMI (Modified Kupperman Menopausal Index) score in order to find factors that affecting the symptoms of menopause and improve the quality of life.

## Methods

### Study design

This cross-sectional study was conducted in 13 cities/autonomous prefectures (including 11 cities and 2 autonomous prefectures) of Gansu Province between August 2016 to May 2017. The sampling design adopted a multi-stage and targeted random sampling method, taking into account the representativeness of this sample. The sampling method for the survey took the following steps. First, according to the population and family income (data from Gansu Province Bureau of Statistics), we divided 13 cities/autonomous prefectures into 9 layers (see the below Table 1). Six districts/counties were randomly selected from each layer, and a total of 54 districts/counties were selected. Second, we divided the 54 districts/counties selected in the first stage into 9 layers and randomly selected a communities/towns from the first, fifth, and ninth layers, and a total of 162 communities/towns were selected. Third, the number of samples in each communities/towns were randomly selected. The survey respondents were organized by the local village committee and Women’s Federation. After excluding those with mental illness and communication illness at baseline, women aged from 40 to 55 years from the selected communities or towns were enrolled in present study.

**Table 1:** Sampling framework

Population Family income	Large	Medium	Small
< 1800 $	1	2	3
1800–3600 $	4	5	6
> 3600 $	7	8	9

### Data collection

Investigations were mainly conducted in the local health center or community health service station. For residents living far away or areas, household visit was applied. Participants that agreed to participate were invited to join a face to face interview with the trained interviewers. All interviewers were required to attend a series of standardized training sessions before commencing interviews.

A questionnaire was used to collect information of baseline characteristics, including age, body weight, height, education, family income, employment status, marital status, menstrual history, reproductive history, lifestyle factors, and chronic diseases.

The menopausal symptoms was measured by mKMI, which has been shown to have a high validity and reliability in Chinese women [19]. It consists of 13 items [20–22], including hot flashes/sweating, paresthesia, insomnia, irritability, depression/suspicion, vertigo, fatigue, arthralgia / myalgia, headache, palpitation, skin formication, sexual life, and urinary infection. Each symptom was divided into four grades according to severity: 0, no symptoms; 1, mild symptoms; 2, moderate symptoms; 3, severe symptoms. Each item has a weighting score. The weighted score for hot flashes and sweating is four; two for the symptom of paresthesia, insomnia, irritability, sexual life abnormality, and urinary tract infection, while one for other symptoms. The total mKMI score is the sum of all items by the weighting factor. The severity of symptoms was defined as: 0–6 (none), 7–14 (mild), 15–30 (moderate) and > 30 (severe).

According to the definition of the Stages of Reproductive Aging Workshop + 10 (STRAW + 10) [23], the standard stages were as follows: (1) premenopause, characterized by minor changes in cycle length, particularly decreasing length of the menstrual cycle, and regular menstrual cycles with ≥ 12 menstruations during the last 12 months; (2) perimenopause, characterized by a variable cycle length (persistent 7 days or more difference in the length of consecutive cycles), missed 2 or more cycles, and an episode of amenorrhea lasting more than 60 days during the last 12 months; and (3) postmenopause, characterized by no menstrual bleeding during the last 12 months (at least 12 months of amenorrhea). Body mass index (BMI) was classified according to the WHO criteria: underweight, BMI < 18.5 kg/m^2^; normal weight, 18.5 ≤ BMI ≤ 23.9 kg/m^2^; overweight, 24 ≤ BMI ≤ 27.9 kg/m^2^; obesity, BMI ≥ 28.0 kg/m^2^.

Data input were done by two different persons separately via the Epidata 3.1 software.

### Statistical analysis

The baseline characteristics, menstrual and menopausal information of enrolled population among the four subgroups of menopausal symptom score were compared via analysis of variance for continuous variables and Chi-square test for categorical variables.

As the response variable (the stratified menopausal symptom score in present study) was ordinally scaled, an ordinal logistic regression model was applied to investigate the potential risk factors of menopausal symptom score (selecting the “none” group as the reference). However, due to ethnicity (Han/Non-Han) did not meet the proportional odds assumption, a partial proportional odds model—one of the ordinal logistic regression models that permitting non-proportional odds for ethnicity– was instead used to well-matched to our data. The factors enrolled in the multivariable model included age at baseline (< 45, 45–50, or ≥ 50 years), BMI, marital status (married/others), occupation (clerical or professional, farmer or manual, or others), family income (< 1800, 1800–3600, or > 3600 $), education (illiterate or elementary school, middle school, high school, or college and above), physical activity (never, 1–3 times/week, 4–6 times/week, or ≥ 7 times/week), history of gynecological disease (yes/no), history of breast disease(yes/no), history of chronic disease(yes/no), age at menarche (< 15 or ≥ 15 years), duration of menstruation (< 5, 5–6 or ≥ 7 days), interval of menstruation (≤ 28 or > 28 days), number of pregnancy (0–1, 2–3, or > 3), duration of breastfeeding (< 12, 12 or > 12 months), menopausal status (pre-, peri-, or post-menopausal), and menopausal hormone therapy (MHT) usage (yes/no). The measurements of those factors were provided in the supplementary file (Additional file 1: Table S1).

A *P* value less than 0.05 was considered statistically significant. All statistical analyses were conducted using SAS software (V. 9.4) (SAS Institute Inc., USA).

## Results

A total of 7500 women aged 40 to 55 years old were initially enrolled, among them, 181 women were excluded due to uncompleted questionnaires (n = 102), refused to fill in the questionnaire (n = 68), or unqualified or wrong answers (n = 11). Eventually, a total of 7319 participants were included.

The result of demographic and socioeconomic characteristics of study population by the mKMI score was shown in Table 2. We found that participants with higher menopausal symptom score are more likely to be older, farmer or manual, higher BMI, higher percentage of having gynecological or chronic disease, lower level of family income, education, and physical activity, but less likely to be Han (ethnic), and married. The menstrual and menopausal information was shown in Table 3. We found that they are more likely to start menarche later (after 15 years old), have longer duration of menstruation, interval of menstruation, number of pregnancy, duration of breastfeeding, and higher percentage of MHT, and be peri- or post-menopausal (Table 3).

**Table 2:** Demographic and socioeconomic characteristics of study population by the mKMI score

	The mKMI score					
	None (0–6)	Mild (7–14)	Moderate (15–30)	Severe (> 30)	Statistics	*P* value
*Age, mean ± SD, years*	45.32 ± 4.02	46.31 ± 4.24	47.77 ± 4.34	49.77 ± 4.14	219.37	< 0.0001
< 45	731 (47.16)	798 (36.89)	748 (24.81)	76 (12.86)	531.127	< 0.0001
45–50	544 (35.10)	834 (38.56)	1112 (36.88)	168 (28.43)		
≥ 50	275 (17.74)	531 (24.55)	1155 (38.31)	347 (58.71)		
*BMI, mean ± SD, kg/m*^*2*^	22.72 ± 2.53	22.85 ± 2.55	23.24 ± 2.90	23.54 ± 3.04	22.25	< 0.0001
< 18.5	58 (3.74)	82 (3.79)	112 (3.71)	27 (4.57)	61.9232	< 0.0001
18.5–23.9	1055 (68.06)	1412 (65.28)	1794 (59.50)	310 (52.45)		
24–27.9	384 (24.77)	611 (28.25)	916 (30.38)	209 (35.36)		
≥ 28	53 (3.42)	58 (2.68)	193 (6.40)	45 (7.61)		
*Ethnic, n(%)*					49.0415	< 0.0001
Han	1420 (91.61)	1993 (92.14)	2685 (89.05)	474 (80.20)		
Others	130 (8.39)	170 (7.86)	330 (10.95)	117 (19.80)		
*Marital status, n(%)*					7.3482	0.0067
Married	1500 (96.77)	2105 (97.32)	2875 (95.36)	567 (95.94)		
Others	50 (3.23)	58 (2.68)	140 (4.64)	24 (4.06)		
*Occupation, n(%)*					161.747	< 0.0001
Clerical or professional	615 (39.68)	675 (31.21)	794 (26.33)	108 (18.27)		
Farmer or manual	522 (33.68)	922 (42.63)	1431 (47.46)	346 (58.54)		
Others	413 (26.65)	566 (26.17)	790 (26.20)	137 (23.18)		
*Family income, n(%)*					31.5785	< 0.0001
< 1800 $	431 (27.81)	622 (28.76)	979 (32.47)	237 (40.10)		
1800–3600 $	541 (34.90)	780 (36.06)	1069 (35.46)	173 (29.27)		
> 3600 $	578 (37.29)	761 (35.18)	967 (32.07)	181 (30.63)		
*Education, n(%)*					266.911	< 0.0001
Illiterate or elementary school	519 (33.48)	896 (41.42)	1550 (51.41)	375 (63.45)		
Middle school	334 (21.55)	501 (23.16)	627 (20.80)	105 (17.77)		
High school	271 (17.48)	330 (15.26)	403 (13.37)	60 (10.15)		
College and above	426 (27.48)	436 (20.16)	435 (14.43)	51 (8.63)		
*Physical activity, n(%)*					11.4011	0.0007
Never	1022 (65.94)	1450 (67.04)	2058 (68.26)	441 (74.62)		
1–3 times/week	127 (8.19)	187 (8.65)	247 (8.19)	31 (5.25)		
4–6 times/week	134 (8.65)	181 (8.37)	284 (9.42)	41 (6.94)		
≥ 7 times/week	267 (17.23)	345 (15.95)	426 (14.13)	78 (13.20)		

The mKMI, the Modified Kupperman Menopausal Index

SD, standard deviation; BMI, body mass index

**Table 3:** Menstrual and reproductive history and other information of study population by the mKMI score

	The mKMI score					
	None (0–6)	Mild (7–14)	Moderate (15–30)	Severe (> 30)	Statistics	*P* value
*Age at menarche, n(%)*					36.0250	< 0.0001
< 15 years	678 (43.74)	875 (40.45)	1085 (35.99)	196 (33.16)		
≥ 15 years	872 (56.26)	1288 (59.55)	1930 (64.01)	395 (66.84)		
*Duration of Menstruation, n(%)*					15.5303	< 0.0001
< 5 days	680 (43.87)	960 (44.38)	1302 (43.18)	227 (38.41)		
5–6 days	608 (39.23)	820 (37.91)	1076 (35.69)	212 (35.87)		
≥ 7 days	262 (16.90)	383 (17.71)	637 (21.13)	152 (25.72)		
*Interval of Menstruation, n(%)*					16.5932	< 0.0001
≤ 28 days	749 (48.32)	1011 (46.74)	1350 (44.78)	224 (37.90)		
> 28 days	801 (51.68)	1152 (53.26)	1665 (55.22)	367 (62.10)		
*Pregnancy time, n(%)*					116.629	< 0.0001
0–1	326 (21.03)	325 (15.03)	348 (11.54)	41 (6.94)		
2–3	1032 (66.58)	1534 (70.92)	2145 (71.14)	414 (70.05)		
> 3	192 (12.39)	304 (14.05)	522 (17.31)	136 (23.01)		
*Duration of breastfeeding, n(%)*					99.9729	< 0.0001
< 12 months	715 (46.13)	874 (40.41)	1087 (36.05)	166 (28.09)		
12 months	453 (29.23)	666 (30.79)	906 (30.05)	173 (29.27)		
> 12 months	382 (24.65)	623 (28.80)	1022 (33.90)	252 (42.64)		
*Menopausal status, n(%)*					706.768	< 0.0001
Premenopause	1066 (68.77)	1202 (55.57)	1098 (36.42)	106 (17.94)		
Perimenopause	282 (18.19)	498 (23.02)	887 (29.42)	179 (30.29)		
Postmenopause	202 (13.03)	463 (21.41)	1030 (34.16)	306 (51.78)		
*MHT, n(%)*					14.1077	0.0002
No	1477 (95.29)	2048 (94.68)	2809 (93.17)	543 (91.88)		
Yes	73 (4.71)	115 (5.32)	206 (6.83)	48 (8.12)		
*Gynecological disease, n(%)*					62.4735	< 0.0001
No	1077 (69.48)	1359 (62.83)	1758 (58.31)	329 (55.67)		
Yes	473 (30.52)	804 (37.17)	1257 (41.69)	262 (44.33)		
*Breast disease, n(%)*					0.0261	0.8716
No	1154 (74.45)	1548 (71.57)	2193 (72.74)	443 (74.96)		
Yes	396 (25.55)	615 (28.43)	822 (27.26)	148 (25.04)		
*Chronic disease, n(%)*					289.862	< 0.0001
No	1461 (94.26)	1930 (89.23)	2454 (81.39)	400 (67.68)		
Yes	89 (5.74)	233 (10.77)	561 (18.61)	191 (32.32)		

MHT, Menopausal hormone therapy; The mKMI, the Modified Kupperman Menopausal Index

The associations of different factors with menopausal symptom score were shown in Tables 4 and 5, respectively. Compared with participants of Han, participants of Non-Han had a higher odds of having higher menopausal symptom score, with effect estimate ranging from 1.21 to 2.29 (Table 4). In multivariable adjusted ordinal logistic regression, we observed that older age, higher BMI, non-married status, longer duration of menstruation (≥ 7 days), number of pregnancy (> 3 times), longer duration of breastfeeding (> 12 months), peri- or post-menopausal status, and MHT was positively associated with increased odds of having a higher menopausal symptom score. While higher level of family income, education, highest level of physical activity, and history of gynecological, breast or chronic disease were associated with lower odds of having a higher menopausal symptom score.

**Table 4:** The effect of ethnic on menopausal symptom score, a cross-sectional study in China

Factor (reference)	Probability of an event	Estimate	Standard error	Wald Chi-square	*P* value	OR (95% CI)
Ethnic (Han)	Pr (Severe)	0.8271	0.1192	48.1804	< 0.0001	2.29 (1.81–2.89)
Ethnic (Han)	Pr (≥ moderate)	0.4379	0.0876	24.9741	< 0.0001	1.55 (1.30–1.84)
Ethnic (Han)	Pr (≥ mild)	0.1947	0.1086	3.2139	0.0730	1.21 (0.98–1.50)

As ethnic does not satisfy the proportional odds assumption, we hereby creates different parameters for ethinic in each response function, that is, ethnic has unequal slopes across the response functions. OR: odds ratio; CI: confidence interval

**Table 5:** Factors associated with menopausal symptom score, a cross-sectional study in China

Factor (reference)	Levels	Estimate	Standard error	Wald Chi-square	*P* value	OR (95% CI)
Age	< 45					1.00 (Ref.)
	45–50	0.2897	0.0543	28.5085	< 0.0001	1.34 (1.20–1.49)
	≥ 50	0.6419	0.0685	87.7373	< 0.0001	1.90 (1.66–2.17)
BMI		0.0264	0.00824	10.2513	0.0014	1.03 (1.01–1.04)
Marital status	Married					1.00 (Ref.)
	Others	0.3852	0.1183	10.5943	0.0011	1.47 (1.17–1.85)
Occupation	Clerical or professional					1.00 (Ref.)
	Farmer or manual	0.0813	0.0718	1.2831	0.2573	1.08 (0.94–1.25)
	Others	0.00385	0.0729	0.0028	0.9578	1.00 (0.87–1.16)
Family income	< 1800 $					1.00 (Ref.)
	1800–3600 $	− 0.0651	0.0562	1.3416	0.2468	0.94 (0.84–1.05)
	> 3600 $	− 0.1035	0.0590	3.0785	0.0793	0.90 (0.80–1.01)
Education	Illiterate or elementary school					1.00 (Ref.)
	Middle school	− 0.3388	0.0618	30.0884	< 0.0001	0.71 (0.63–0.80)
	High school	− 0.5871	0.0775	57.3372	< 0.0001	0.56 (0.48–0.65)
	College and above	− 0.8246	0.0901	83.7898	< 0.0001	0.44 (0.37–0.52)
Physical activity	Never					1.00 (Ref.)
	1–3 times/week	0.1227	0.0836	2.1531	0.1423	1.13 (0.96–1.33)
	4–6 times/week	0.1773	0.0823	4.6377	0.0313	1.19 (1.02–1.40)
	≥ 7 times/week	− 0.1533	0.0645	5.6500	0.0175	0.86 (0.76–0.97)
Age at menarche	< 15 years					1.00 (Ref.)
	≥ 15 years	0.00505	0.0471	0.0115	0.9146	1.01 (0.92–1.10)
Duration of Menstruation	< 5 days					1.00 (Ref.)
	5–6 days	0.0201	0.0496	0.1636	0.6858	1.02 (0.93–1.12)
	≥ 7 days	0.1768	0.0606	8.5210	0.0035	1.19 (1.06–1.34)
Interval of Menstruation	≤ 28 days					1.00 (Ref.)
	> 28 days	0.0153	0.0450	0.1160	0.7335	1.02 (0.93–1.11)
Pregnancy time	0–1					1.00 (Ref.)
	2–3	0.1689	0.0670	6.3618	0.0117	1.18 (1.04–1.35)
	> 3	0.3814	0.0834	20.9303	< 0.0001	1.46 (1.24–1.72)
Duration of breastfeeding	< 12 months					1.00 (Ref.)
	12 months	0.000538	0.0553	0.0001	0.9922	1.00 (0.90–1.12)
	> 12 months	0.1316	0.0574	5.2595	0.0218	1.14 (1.02–1.28)
Menopausal status	Premenopause					1.00 (Ref.)
	Perimenopause	0.7530	0.0563	178.7927	< 0.0001	2.12 (1.90–2.37)
	Postmenopause	0.9270	0.0662	196.0497	< 0.0001	2.53 (2.22–2.88)
Gynecological disease		− 0.3821	0.0470	66.0550	< 0.0001	0.68 (0.62–0.75)
Breast disease		− 0.2354	0.0534	19.4351	< 0.0001	0.79 (0.71–0.88)
Chronic disease		− 0.6737	0.0665	102.5331	< 0.0001	0.51 (0.45–0.58)
MHT (yes)		0.3312	0.0964	11.8077	0.0006	1.39 (1.15–1.68)

Factors in Table 5 satisfies the proportional odds assumption of ordinal logistic regression, then they have equal slopes across the response functions

BMI, body mass index; MHT, Menopausal hormone therapy; OR, odds ratio; CI, confidence interval; Ref, reference

## Discussion

We found that participants with higher menopausal symptom score are more likely to be older, farmer or manual, higher BMI, higher percentage of having gynecological or chronic disease, lower level of family income, education, and physical activity, but less likely to be Han (ethnic), and married. We also found that they are more likely to start menarche later (after 15 years old), have longer duration of menstruation, interval of menstruation, number of pregnancy, duration of breastfeeding, and higher percentage of MHT, and be peri- or post-menopausal.

### Racial/ethnic

Present study found that racial/ethnic affects the prevalence of the menopausal symptoms, which is in line with the result of the Study of Women’s Health across the Nation (SWAN) study [24–26]. Although China is a multi-ethnic country and Gansu Province has unique ethnic minorities, few researches on the status of menopausal women was conducted in these areas. Some studies reported that the prevalence and severity of menopausal symptoms vary among perimenopausal and postmenopausal women in different racial groups [27–29]. Similarly, we found a statistical significance among the severity of menopausal syndrome in the different ethnic groups (*P* < 0.05) (Table 4). In ethnic minority areas, low level of economy, shortage of educational resources, poor-equipped health systems and medical resources are all contributors for the low quality of ethnic minority women's life. Minorities have formed their unique culture, for example, Hui and Tibetan people are particularly fond of eating beef and mutton and usually live at high altitudes, where ultraviolet light is strong and oxygen is scarce. We therefore speculate that diet, natural environment, social environment played roles in the type and severity of menopausal symptoms which requires further research.

### Physical activity

Physical activity may play a protective role in attenuating climacteric symptoms [30–32] and improve the quality of life of the middle-aged women [33]. In Ronit et al*.* study, regular exercise was negatively correlated with the severity of the menopausal syndrome, where middle-aged women with higher physical activity had less severe menopausal symptoms [34]. Two randomized controlled trial studies in Chinese showed that 12-week exercise therapy effectively improves the symptoms [27, 35]. In our study, we found that people who exercised less than 7 times per week had more severe menopausal symptoms than those who never exercised, while those who exercised more than 7 times had less severe menopausal symptoms. This contradictory result may be explained by two reasons. Firstly, we assumed that participants who exercised more than 7 times per week (regular exerciser) may potentially represent a distinct subgroup population, compared to those who exercised less than 7 times per week. For the latter group, searching for exercising may be a consequence of the menopausal symptoms. The positive association observed in the participants who exercised less than 7 times per week therefore might be caused by reversion causation. However, we also cannot rule out the possibility that this contradictory result was caused by chance. Validation studies from independent populations are needed. Around 40% women in our study participated in the square dancing, which is especially favored by the middle-aged and old people [36]. Additionally Square dancing not only positively affects fitness and health, but also helps mediate participants’ emotions and improve interpersonal relationships.

### Educational level

The relationship between education level and menopausal symptoms was varied in different studies, some studies have shown that education was effective in reducing the early symptoms of menopause [37, 38], while a study in the southeast China found that compared with women with only a primary school education or below, women who received secondary education were 1.55-fold more likely to have menopause syndrome [18]. On the contrary, in present study, we observed that women with poor educational background have higher mKMI score, which is consistent with the results of Castelo-Branco et al*.* [39]. Yim et al*.* however reported that Korean women who had not completed high school had fewer symptoms [2]. Women with higher levels of education and socioeconomic status were more concerned with their health statuses and were able to have better access to health care plans based on their symptoms rather than putting up with physical discomfort [33]. Therefore, more studies are warranted to clarify the relationship between educational background and menopausal symptoms.

### Living area

Some studies showed that women who lived in suburban or rural areas were less likely to suffer from menopause syndrome than women who lived in urban areas [18, 40]. In contrast, we found that farmers in rural areas had severe menopausal symptoms compared with clerical or professional Chinese urban women. Women in rural area tend to have lower education levels and may lack of knowledge on reproductive health; additionally, health conditions was lag behind in those cities. Previous studies have shown that the universal perception of menopause is: it is the adverse events during the menopausal transition, which need to be tolerated and will vanish over time [34]. This perception might be a major reason for the delays in menopausal health care seeking [41].

### Marital status

Gibbs et al. [42] and Gao et al. [43] found that unmarried, divorced, widowed, or other marital status women had lower subjective satisfaction during perimenopausal period and had increased risk of depression compared with married women. In our study, women who did not have a partner can lead to more severe menopausal symptoms, which had the same result as a study in South Korea [44]. Good marital status can give women higher quality of sexual life and conjugal relations, promote physical and psychological health, and provide social support to help them deal with stressful events which may result in depression [43].

There was an increasing trend in both the prevalence and the severity of most menopausal symptoms as menopause progressed. By utilizing logistic regression analyses and adjusting confounding factors, we identified gynecological disease, chronic disease and breast disease were associated with the occurrence of menopausal symptoms. Many menopausal symptoms including somatic, psychological and urogenital symptoms may interact with symptoms of other diseases. Further studies are needed regarding the effects of chronic disease in menopausal symptoms. Our study also found that pregnancy times were associated with the severity of menopausal symptoms which is consistent with the results of previous studies [45].

Our findings indicated that women lived in the Northwest China have little knowledge about menopause and self-care. Many women did not know they had already entered the menopausal transition, and thought that the various aspects of physical emotional discomfort were due to pressure from work or family others. They were more likely to think that menopause is a natural phenomenon and chose to tolerate and ignore the symptoms, which may affect their healthcare-seeking behavior. Our finding was similar to the results from previous studies, in which women would not seek treatment unless they experienced multiple or severe symptoms [46, 47].

The middle-aged women will be confronted with social and psychological pressures during this special transitional stage, care and help from family and society therefore will eliminate their pressures and reduce the occurrence of menopausal symptoms to some extent. Regular physical activity may alleviate menopausal symptoms as well. Besides, more fundamental researches and epidemiological surveys should be conducted to provide more evidence for clinical practice.

### Limitation

There are several potential limitations in our study. First, the cross-sectional study design was prone to potential selection and information biases. For example, all variables was assessed by self-reported questionnaire, which could lead to misclassification to some extent, the findings therefore were only suggestive and cannot prove causality. Second, due to the ethnicity did not meet the proportional odds assumption of the ordinal logistic regression; we were therefore unable to explore the interactions between ethnicity and other covariables. Third, laboratory data on sex hormone levels, including AMH (anti-Mullerian hormone), serum estrogen, and FSH levels, were not available. Fourth, as we investigated this association in one regional in China, the generalization of our findings to other areas and ethnicities should be cautious.

## Conclusion

In the present study, we found that age, marital status, racial/ethnic, physical activity, educational level, and living area may be associated with menopausal symptoms. A better understanding of these factors may help to reduce the effect of menopausal symptoms on the quality of life of the middle-aged women. The middle-aged women tend to be confronted with social and psychological stress and negative life events. In this special stage, care and help from family and society will eliminate their stress and reduce the occurrence of menopausal symptoms. Moreover, more relevant fundamental researches and epidemiological surveys should be conducted to provide more powerful evidence for clinical practice.

## Supplementary Information

**Additional file 1: Table S1**. Measurements of variables.

## Data Availability

All data generated or analysed during this study are included in this published article.
