# Supplementary material for: Severity and factors of menopausal symptoms in middle-aged women in Gansu Province of China: a cross-sectional study
Source: BMC Womens Health. 2021 Dec 8;21:405. doi: 10.1186/s12905-021-01531-x (PMC8653527; doi:10.1186/s12905-021-01531-x)
Supplement: Supplementary file 1 — Additional file 1: Table S1. Measurements of variables. [file 12905_2021_1531_MOESM1_ESM.pdf]

Table S1 Assessments of variables.

Variables	Methods of assessment	Details of assessments
Age	How old are you?	Fill in the numbers
BMI	Measure height and weight	Calculated as weight (kg)/ height (m2)
Ethnic	What is your ethnic?	Fill in the specific contents
Marital status	What is your marital status?	Fill in the specific contents
Occupation	What is your job?	Fill in the specific contents
Family income	How much do your family earn in one year?	< 1800 \$<br>1800-3600 \$<br>> 3600 \$
Education	What is your educational level?	Illiterate or Elementary school<br>Middle school<br>High school<br>College and above
Physical activity	How often do you exercise per week?	Never<br>1-3 times/week<br>4-6 times/week<br>≥ 7 times/week
Age at menarche	What was the age at menarche?	Fill in the specific contents
Duration of Menstruation	How long does menstruation last each cycle?	< 5 days<br>5-6 days<br>≥ 7 days
Interval of Menstruation	What is the interval of menstruation?	≤ 28 days<br>> 28 days
Pregnancy time	How many pregnancy times?	Fill in the specific contents
Duration of breastfeeding	Breastfeeding or not, if yes, how long?	< 12 months

		12 months<br>> 12 months
Menopausal status	Is your menstruation regular? If not, please select	Premenopause: decreasing length of the menstrual cycle, and regular menstrual cycles with ≥12 menstruations during the last 12 months;<br>Perimenopause: a variable cycle length (persistent 7 days or more difference in the length of consecutive cycles), missed 2 or more cycles, and an episode of amenorrhea lasting more than 60 days during the last 12 months;<br>Postmenopause: no menstrual bleeding during the last 12 months
MHT	Do you have ever used MHT?	No<br>Yes
Gynecological disease	Do you have any of the following gynecological diseases?	none<br>cervical erosion<br>uterine myoma<br>cervical intraepithelial neoplasias<br>cervical cancer<br>endometrial carcinoma<br>ovarian cysts<br>ovarian cancer<br>others
Breast disease	Do you have any of the following breast diseases?	none<br>hyperplasia of mammary glands<br>fibroadenoma of breast<br>breast cancer

		others
Chronic disease	Do you have any of the following chronic diseases?	none
		heart disease
		hypertensive
		hyperlipaemia
		diabetes
		hepatitis
		tuberculosis
		appendicitis
		others

Abbreviations: BMI: body mass index. MHT Menopausal hormone therapy.
